# Interaction between *SLC6A4* promoter variants and childhood trauma on the age at onset of bipolar disorders

**DOI:** 10.1038/srep16301

**Published:** 2015-11-06

**Authors:** B. Etain, M. Lajnef, A. Henrion, A.A. Dargél, L. Stertz, F. Kapczinski, F. Mathieu, C. Henry, S. Gard, J. P. Kahn, M. Leboyer, S. Jamain, F. Bellivier

**Affiliations:** 1 Inserm U955, Equipe Psychiatrie Translationelle, Créteil, 94000, France; 2 AP-HP, Hôpitaux Universitaires Henri Mondor, DHU PePSY, Pôle de Psychiatrie et d’Addictologie, Créteil, 94000, France; 3 Université Paris Est, Faculté de Médecine, Créteil, 94000, France; 4 Fondation Fondamental, Fondation de Coopération Scientifique, Créteil, 94000, France; 5 Laboratório de Psiquiatria Molecular, Instituto Nacional de Ciência e Tecnologia – Translacional em Medicina (INCT), Hospital de Clínicas de Porto Alegre, Programa de Pós-Graduação em Psiquiatria, Universidade Federal do Rio Grande do Sul, Porto Alegre, RS, Brazil; 6 UT Center for Molecular Psychiatry, Department of Psychiatry and Behavioral Sciences, University of Texas Health Science Center, Houston, TX, USA; 7 Laboratório de Psiquiatria Molecular, Instituto Nacional de Ciência e Tecnologia – Translacional em Medicina (INCT), Hospital de Clínicas de Porto Alegre, Universidade Federal do Rio Grande do Sul, Porto Alegre, RS, Brazil; 8 INSERM UMR-S958, Université Paris 7, Faculté de Médecine de Villemin, Paris, France; 9 Pôle 3-4-7, Service de Psychiatrie Adulte, Centre Expert Trouble Bipolaire, Hôpital Charles Perrens, Bordeaux, France; 10 Service de Psychiatrie et Psychologie Clinique, CHU de Nancy, Hôpitaux de Brabois, Vandoeuvre Les Nancy, Nancy, France; 11 APHP, Hôpital Fernand Widal, Pôle Addictologie-Toxicologie-Psychiatrie, Paris, France; 12 Université Paris-7, Paris, France

## Abstract

Age at onset (AAO) of bipolar disorders (BD) could be influenced both by a repeat length polymorphism (5HTTLPR) in the promoter region of the serotonin transporter gene (SLC6A4) and exposure to childhood trauma. We assessed 308 euthymic patients with BD for the AAO of their first mood episode and childhood trauma. Patients were genotyped for the 5HTTLPR (long/short variant) and the rs25531. Genotypes were classified on functional significance (LL, LS, SS). A sample of 126 Brazilian euthymic patients with BD was used for replication. In the French sample, the correlation between AAO and trauma score was observed only among ‘SS’ homozygotes (p = 0.002) but not among ‘L’ allele carriers. A history of at least one trauma decreased the AAO only in ‘SS’ homozygotes (p = 0.001). These results remained significant after correction using FDR. Regression models suggested an interaction between emotional neglect and ‘SS’ genotype on the AAO (p = 0.009) and no further interaction with other trauma subtypes. Partial replication was obtained in the Brazilian sample, showing an interaction between emotional abuse and ‘LS’ genotype on the AAO (p = 0.02). In conclusion, an effect of childhood trauma on AAO of BD was observed only in patients who carry a specific stress responsiveness-related *SLC6A4* promoter genotype.

Bipolar disorder (BD) is a heterogeneous and complex disorder with a high heritability (close to 60%) and interactions with environmental risk factors (Lichtenstein *et al.*, Lancet, 2009). In order to identify homogeneous subforms of the disorder, candidate symptom such as age at onset (AAO) of the disorder have been suggested^1^,^2^. Early AAO has repeatedly been demonstrated to be a valid clinical marker of a subgroup of BD patients with a high familial risk that is coupled to increased comorbid medical and psychiatric disorders and poor prognosis^1^,^2^,^3^. Indeed, early BD onset associates with prototypic clinical characteristics, such as more frequent psychotic symptoms during mood episodes, more manic and mixed episodes, and more frequent suicidal behavior. Elevated frequencies of comorbid psychiatric conditions such as panic disorder, conduct disorder, alcohol and/or substance misuses, attention deficit/hyperactivity disorder and of severe medical illnesses such as cardiac, gastro-intestinal, and neurological disorders are also observed in patients with an early AAO of BD^1^,^2^,^4^,^5^. An early onset also associates with a greater severity and a poorer long-term outcome, as evidenced by chronicity and it potential resistance to mood stabilizers, such as lithium salts^1^,^6^. Several studies have shown AAO to be a heritable clinical characteristics in families affected by BD^1^,^7^,^8^, although one study failed to replicate these findings^9^. This intrafamilial resemblance suggests that AAO is determined by common familial factors of genetic and/or environmental origins. However, these factors remain to be clearly identified.

Childhood adverse events may influence the AAO of BD. Two recent reviews of the literature highlighted that the global level of trauma or nonspecific early abuses are associated with an earlier AAO, perhaps especially sexual and physical abuses^10^,^11^,^12^. However, the methodological quality of the reviewed studies has been relatively poor, primarily due to small sample sizes and a lack of standardized assessment of BD diagnosis and childhood trauma. To overcome these methodological difficulties, we recently studied the influence of childhood trauma, using the Childhood Trauma Questionnaire (CTQ), on the clinical expression of BD in a large sample of 587 BD patients from France and Norway^13^. We found that AAO was lower in patients who had experienced various trauma subtypes, including sexual abuse, emotional abuse, emotional neglect and physical neglect. We also observed a significant dose relationship between childhood abuse and earlier AAO, with AAO being significantly lower in BD patients exposed to more types of abuse. Consequently, childhood trauma(s) are suggested to influence the AAO in BD.

Genetic factors can also influence AAO in BD. Three independent genetic association studies have concluded that a repeat length polymorphism (5HTTLPR) in the promoter region of the *SLC6A4* gene (OMIM: 182138) encoding the serotonin transporter, can influence the AAO in BD. An increased presence of the short allele (‘s’) of the 5HTTLPR has been observed in BD patients with an early AAO^14^. Similarly, in a sample of BD patients of French origin, carriers of the 5HTTLPR ‘ss’ genotype tended to have an earlier AAO^15^. Finally, a mixture regression analysis also suggested the influence of 5HTTLPR on the AAO in BD^16^. Overall, these findings suggest that 5HTTLPR could play a role in the modulation of BD AAO. Previous results in a sample of 136 patients with BD suggested that the 5-HTTLPR significantly modulated the relationship between early life stress and age at onset of BD^17^.

On the basis of such data, this study investigated the interaction between *SLC6A4* promoter variants and CTQ (total score and subtypes of abuses and neglects) on the AAO of BD.

## Materials and Methods

### Samples

The initial sample consisted of euthymic patients with BD type I, II or not otherwise specified (NOS) who were recruited from three psychiatric departments in France (Paris/Créteil, Bordeaux and Nancy). Patient inclusion criteria were: aged over 18 years; having a diagnosis of BD according to DSM-IV criteria^18^; being Caucasian; and clinically normothymic at the time of inclusion (i.e. having a Montgomery Asberg Depression Rating Scale score^19^ and a Mania Rating Scale score^20^ below five as well as no major mood episodes in the last three months). Patients were interviewed using the French version of the Diagnostic Interview for Genetic Studies (DIGS)^21^, providing lifetime DSM-IV axis I diagnoses^18^. The AAO of BD was determined retrospectively and was defined as the age at which a patient first met DSM-IV criteria for a major depressive or (hypo)manic episode according to clinical information collected with the DIGS. This sample was a subsample of the one published in 2013 that included 418 patients from France and 169 patients from Norway^13^. For this study, only patients from France were included and the inclusion was based only on availability of DNA samples. No other exclusion criteria were used (in particular clinical ones). Written informed consent was obtained from all participants. This study was approved by the medical ethics committee of the French << Comité de Protection des Personnes (CPP) >> (IDRCB2008_AO1465_50 VI—Pitié Salpêtrière 118-08) and carried out in accordance with the approved guidelines.

The replication sample consisted of patients with BD (type I, II or NOS) who were recruited from the outpatient Bipolar Disorders Program of Hospital de Clínicas de Porto Alegre (HCPA) in Brazil. Patients were diagnosed according to the criteria of the Diagnostic and Statistical Manual of Mental Disorders, 4th edition (DSM-IV-TR)^18^. The confirmation of the diagnosis was established by board certified psychiatrists using the Structured Clinical Interview for DSM-IV Axis I Disorders (SCID-I)^22^. The inclusion criteria of the patients were age from 18 to 65 years old, BD diagnosis according to DSM-IV- TR criteria, and absence of an acute mood episode during the previous month assessed by means of the Hamilton Rating Scale Score (HDRS)^23^ and the Young Mania Rating Scale (YMRS)^24^ <15. The AAO of BD was determined retrospectively and was defined as the age at which a patient first met DSM-IV criteria for a major depressive or (hypo)manic episode according to clinical information collected with the SCID-I. The research ethics committee of HCPA approved the study protocol. All subjects provided written informed consent before their inclusion in the study.

### Assessment of childhood affective trauma

Childhood traumatic events were recorded using the CTQ, a 28-item self-report questionnaire^25^. The CTQ yields a total score and five subscale scores for emotional and physical neglect, as well as emotional, physical and sexual abuses. The CTQ total score was used as a continuous variable assessing trauma severity. Based on the guidelines and cut-off values proposed by Bernstein and Fink^26^, we used the scores for each subscale to determine whether each kind of trauma was present (mild, moderate or severe) or absent. The French and the Portuguese validated versions were used in this study^27^,^28^.

### Genotyping

DNA was prepared from peripheral blood leukocytes or B-lymphoblastoïd cell lines by standard procedures. The genotyping of the 5-HTTLPR, rs25531 as well as the triallelic intron 2 VNTR (Stin2) (not used in this study) were performed by triplex polymerase chain reaction (PCR) followed by restriction endonuclease digestion as described by^29^. Briefly, the amplification reaction was performed using three couples of oligonucleotide primers in a final volume of 20 μl containing 20 ng of genomic DNA and using a 0.5 unit of MyTaq™ DNA Polymerase (Bioline, London, UK). After an initial denaturation at 95 °C for 15 min, the amplification cycles consisted of 35 cycles of denaturation at 95 °C for 30 s, annealing at 65.5 °C for 90 s and elongation at 72 °C for 1 min. Subsequently, 8 μl of amplification products were digested with 5U of HpaII (New England Biolabs, Ipswich, MA, USA), in a 20 μl reaction assay containing 1X NEBuffer 1 and 1X BSA at 37 °C for 1 h. Digested and non-digested PCR products were loaded on 3% agarose gel in 1X TBE. The short ‘s’ and the long ‘l’ alleles (as well as the Stin2.9, Stin2.10 and Stin2.12 alleles not used in this study) were determined from the non-digested PCR products with fragments of 469 bp, 512 bp, 250 bp, 267 bp and 300 bp respectively. The rs25531 A/G polymorphism was determined after HpaII digestion according to the following fragment lengths (LA: 512 bp, LG: 402 + 110 bp, SA: 469 bp and SG: 402 + 67 bp). The more common LA allele is associated with the reported higher basal activity, whereas the less common LG allele has transcriptional activity no greater than the S. Therefore, it is suggested that in tests of association the LG alleles should be analyzed along with the S alleles.

### Statistical analyses

The two samples (French and Brazilian) were compared using non-parametric test (Mann-Whitney, Chi-square tests) for descriptive analysis. Since AAO and CTQ scores were assumed not to have a normal distribution, non-parametric tests were used. In this case, Medians and Median Absolute Deviations (MAD) were given. We tested whether the genotype distributions were in Hardy–Weinberg equilibrium.

Linear regression analyses were performed to examine the relationship between AAO, *SLC6A4* promoter genotypes and CTQ subtypes as the predictive variables, and the interactions between CTQ subtypes and genotypes was used as the confounding factors. The model was AAO transformed ~ Genotypes(LL versus LS versus SS) + CTQ subtype + Genotypes × CTQ subtype. AAO was reciprocal square root transformed in the French sample and square root transformed in the Brazilian sample to fulfill the normality assumption required by the parametric procedure.

We corrected for multiple testing in the French sample, using a FDR (False Discovery Rate) with a p-value = < 0.002 being significant. Statistical analyses were performed with the STATA package (version 12)^30^.

## Results

### Description of the French sample

The French sample comprised 308 patients with BD (129 men and 179 women; mean age at interview 43.25 ± 12.6 years old; 229 BD type I, 76 BD type II and 3 BD type NOS). All patients completed the CTQ. Three hundred patients were successfully genotyped for the l/s variant (5HTTLPR) and for the rs25531 (8 DNA failed to be amplified by PCR). The *SLC6A4* promoter variants genotypic frequencies were in Hardy-Weinberg equilibrium (p = 0.48).

### Influence of the SLC6A4 genotypes on the AAO and on the CTQ total score

The mean AAO of BD was 24.72 ± 9.9 years (median = 22, MAD = 7, range 10–67) and the mean CTQ total score was 42.05 ± 12.9 (median = 39, MAD = 7, range 25–99). AAO and CTQ total score did not differ according to genotypes (see Table 1).

### Association between AAO and non-specific trauma according to SLC6A4 genotypes

We first tested for an effect of *SLC6A4* genotypes on non-specific level of trauma (i.e. CTQ total score, then a positive history of at least one trauma subtype).

The AAO and the CTQ total score were negatively correlated for the whole sample of patients (rho = − 0.16 p = 0.004). However, this correlation was mainly due to patients carrying the ‘SS’ genotype (rho_’SS’_ = − 0.32 p = 0.002) and the ‘LS’ genotype (rho_’LS’_ = − 0.17 p = 0.03) with no correlation observed for those patients carrying the ‘LL’ genotype (rho_’LL’_ = − 0.02 p = 0.88).

**Figure 1:**
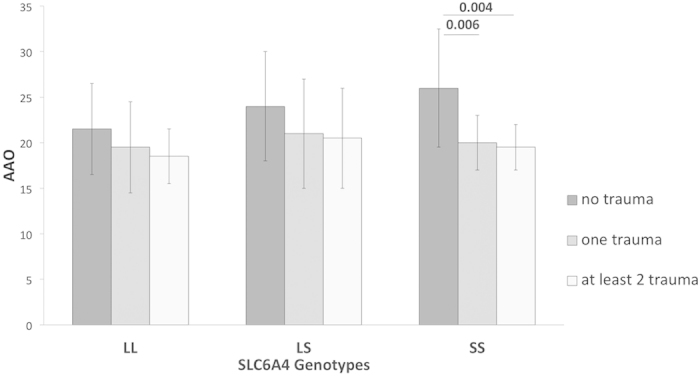
Medians and Median Absolute Deviations of AAO according the absence/presence of trauma and the *SLC6A4* genotypes. Legend: *SLC6A4* (serotonin transporter gene).

We then considered the effect of the absence/presence of any trauma sub-type (regardless of the subtype considered) on the AAO according to genotypes. AAO decreased in the presence of trauma (one or two and more trauma subtype(s)) in the ‘SS’ subgroup (Wilcoxon rank test: p = 0.001) and to a lesser extend in the ‘LS’ subgroup (p = 0.04) but not in the ‘LL’ subgroup (p = 0.44) (see Fig. 1 for details).

### Multivariate regression analyses using CTQ subtypes

Since not all subtypes of trauma are supposed to have equal effects on the AAO with emotional and sexual trauma having possibly a preferential effect^13^, we tested the following model AAO transformed ~ Genotypes(LLvsLSvsSS) + CTQ subtype + Genotypes × CTQ subtype for each trauma subtype. Transformed AAO (1/sqrt(AAO)) fulfilled the normality assumption (Shapiro-Wilk W = 0.24; p > 0.05).

No significant result was observed when studying physical neglect, physical abuse and emotional abuse. We found an interaction between emotional neglect and ‘SS’ genotype on the AAO (p = 0.009) with a trend for an interaction also between emotional neglect and ‘LS’ genotype on the AAO (p = 0.09) in the same model (see Table 2). We found an association between sexual abuse on the AAO (p = 0.02) with no interaction between this subtype of trauma and genotypes (data not shown).

A power calculation with 5 predictors in the regression models showed that our sample size (n = 300) had a power of 0.76 to identify an effect size of 0.04 (small according to the Cohen’s conventional criteria). A sample size of n = 326 would have been required to reach a power of 0.80 and to identify an effect size of 0.04.

### Replication study

The Brazilian sample consisted of 126 patients with BD (23 men and 103 women; mean age at interview 43.03 ± 10.7 years old; 84 BD type I, 40 BD type II and 2 BD type NOS). All patients completed the CTQ with a mean total score of 52.39 ± 20.4. One hundred and twenty two patients were genotyped for the 5HTTLPR l/s variant and for the rs25531 (4 DNA failed to be amplified by PCR). The distribution of genotypes were the following : 30 patients with ‘LL’ genotype, 63 with ‘LS’ genotype and 29 with ‘SS’ genotype. The *SLC6A4* promoter variants genotypic frequencies were in Hardy-Weinberg equilibrium (p = 0.82).

The replication sample differed from the initial one for gender distribution (p < 0.001), BD subtypes distribution (p = 0.02) and CTQ total score (p < 0.001); this corresponded to a Brazilian sample consisting of more females, more BD type II and with a higher CTQ total score. The two samples were similar for age at interview (p = 0.99), age at onset (p = 0.59) and genotypes distribution (p = 0.27).

Multivariate regression analyses used transformed AAO (sqrt(AAO)) that fulfilled the normality assumption (Shapiro-Wilk W = 0.98; p > 0.05). No significant result was observed when studying physical neglect, physical abuse, nor sexual abuse. We found an interaction between emotional abuse and ‘LS’ genotype on the AAO (p = 0.02) and a trend for an interaction between emotional neglect and ‘LS’ genotype on the AAO (p = 0.08). No interaction was found with ‘SS’ genotype in both models (see Tables 3 and 4).

A power calculation with 5 predictors in the regression models showed that this replication sample (n = 122) had a power of 0.34 to identify an effect size of 0.04.

## Discussion

The identification of factors determining the variability of the AAO in BD is of crucial importance as an early AAO consistently associates with a more severe clinical profile and a poor prognosis^1^,^2^,^4^. The identification of risk factors for an earlier AAO could therefore guide both preventive and more personalized strategies in those at risk of developing BD. Both genetic and environmental determinants are thought relevant to AAO heterogeneity in BD^1^. In this study, we provided results suggesting an interaction between *SLC6A4* promoter variants and emotional trauma on the AAO of BD. In the French sample AAO significantly decreased in the presence of trauma mainly in ‘SS’ carriers, this being significant after correction for multiple testing. Regression analyses suggested (although only nominally significant) that emotional traumatic events might be of special interest when studying environmental risk factors associated with an early occurrence of BD. In the replication sample, the results were partly replicated with some nominal signals in favor an interaction between *SLC6A4* promoter variants and emotional trauma on the AAO.

As previously suggested by Benedetti *et al.*^17^, this study suggests a gene/environment interaction influencing the AAO of BD and follows several previous findings suggesting that each factor separately decreases the AAO^10^,^11^,^13^,^14^,^15^,^16^. Our data can be placed within gene/environment models of psychiatric conditions, where environmental factors can represent major risk factors for the development of mental disorders, but that their influence is modulated by the presence of susceptibility genetic variants^31^. Our data highlight a number of processes relevant to the onset and course of BD.

Firstly, our results are consistent with previous pathophysiological data obtained in various experimental models, including knockout mice, stress-reared rhesus macaques and human functional brain imaging^32^. The serotonin transporter genotype has been shown to be associated with differences in physiological responsiveness to stress conditions in these models, with a relative loss of serotonin transporter gene function being associated with greater vulnerability to environmental stress in all species tested^33^,^34^. 5HTTLPR not only regulates serotonergic brain transmission, but carriers of the short allele also display various physiological abnormalities such as increased amygdala reactivity and fear conditioning, as well as alterations in hypothalamo-pituitary-adrenal axis reactivity^33^. Therefore, short allele carriers might present with a decreased ability to appropriately regulate stress responses, thereby lowering the threshold for the occurrence of a first BD episode.

Second, the mechanisms by which childhood traumatic events decrease the AAO in BD remain unclear. It is likely that this is mediated through the induction of a cascade of neurobiological and neuroendocrine events. Previous studies have suggested that childhood trauma can have long-lasting effects on the catecholamine response to psychological stress^35^,^36^ and induce hyper reactivity of the corticoid-releasing factor system^37^,^38^,^39^, as well as altering the structure and function of the medial prefrontal cortex and hippocampus^40^. Although the effects of such trauma are probably not specific to BD, our data suggests that a history of childhood trauma in BD produces long-term disturbances of neurobiological mechanisms that are required to regulate stress and stress-resilience^41^, with this then interacting with alterations in genetic regulation of the serotonergic system. Such physiological changes may lower the threshold for the onset of BD, including via sensitization to other environmental triggering factors.

This causal link (i.e. a greater exposure to trauma leading to an earlier AAO) is not the sole interpretation although above mentioned arguments favor it. Other interpretations that consider ontology, parental psychopathology/psychiatric disorders or gene/environment correlations should also be considered. Patients with a ‘SS’ genotype could present with a more pronounced emotional bias toward negative stimuli^42^ and might thus ‘over-report’ emotional trauma. Second, patients with a ‘SS’ genotype might also present with specific neural defects (such as amygdala over-activation)^43^ leading them to more sensitivity to negative events and thus, here again, to ‘over-report’ emotional trauma (this hypothesis is probably linked to the emotional attention bias one). In both cases, at-risk genotypes could confer greater attention to and/or more reactivity to emotional trauma. Third, patients with a ‘SS’ genotype might present with other psychiatric disorders (mainly externalized ones, such as attention deficit and hyperactivity disorder, conduct disorder, impulsivity)^23^,^24^,^44^ that are associated with more behavioral problems during childhood/adolescence and thus greater exposure to hard discipline. Although not definitively excluded, these hypotheses favoring a gene/environment correlation are not sustained in our sample since no difference in CTQ scores was observed across genotypes.

The design of this study has several limitations. First, the retrospective assessment of traumatic events during childhood may be influenced by uncontrolled recall bias^45^. However, previous reviews have suggested that retrospective self-reports concerning childhood abuse are more likely to be biased toward underreporting than exaggeration^46^,^47^. Second, the retrospective nature of the AAO assessment also provides a source of potential recall bias, although previous data in this area suggests that this is unlikely^48^,^49^. Third, current mood state may lead bipolar patients to under- or over report histories of childhood trauma. We did not adjust CTQ scores for the presence of residual mood symptoms, which we presumed to be very minor, given that we assessed patients during remission periods. As such, potential mood determined biases in early trauma reporting are minimized. Fourth, we did not extensively investigate the genetic structure of *SLC6A4*, focusing only on the promoter region, nor epigenetic mechanisms. Finally, after correction for multiple testing, we also did not reach significance for in-depth investigations of trauma subtypes. We also were not able to totally replicate our results. This issue was possibly related to sample size and reduced power in the replication sample and incomplete comparability between samples. For example, the number of patients with a ‘SS’ genotype in the replication sample was small (n = 29) and had probably reduce our ability to identify an interaction with this particular genotype. Nevertheless, trends for interaction in the larger ‘LS’ genotype group were suggested as shown in the initial sample. Given these limitations, our results will definitively require replication in independent and larger samples of patients with BD. Indeed, the results of gene/environment interaction studies in psychiatry have been a source of controversy, often being hard to replicate or providing mixed results, as in the case of the interaction between 5HTTLPR, stressful life events and the risk of unipolar depression^33^,^50^,^51^,^52^,^53^.

Further research is also required on the genetic and environmental factors that modulate the AAO in BD, which is likely to be multifactorial and determined by many genes with small effect sizes or other variants not measured well by SNP arrays, such as rare alleles or copy number variations^54^,^55^,^56^. Future studies then should investigate other candidate genes that have been suggested to modulate the AAO such as those encoding the apolipoprotein E^57^, the dopamine receptors DRD1, DRD2 and DRD3^58^,^59^,^60^, CACNA1C^61^, the brain derived neurotrophic factor (possibly in interaction with sexual abuse)^62^,^63^, the catechol-O-methyltransferase^64^, the glucocorticoid and mineralocorticoid receptors^65^, HTR2A and HTR2C^16^, Period3^66^, the glycogen synthase kinase 3-beta^67^,^68^,^69^ or SNAP25^70^,^71^. Interestingly, we recently demonstrated the interaction between a variant of the Toll-like receptor gene (*TLR2*) and sexual abuse on the AAO of BD^72^. Several other environmental factors can also contributed to the modulation of the AAO such as solar insolation, light exposure^73^,^74^,^75^ or exposure to stimulants^76^, as well as antidepressants^77^. Among investigated environmental factors that modulate the AAO of BD, the role of early cannabis misuse has been widely proposed^4^,^78^,^79^,^80^,^81^,^82^. The picture is further complicated by the possibility of significant additive effects evident between cannabis abuse and childhood abuse on an earlier AAO in BD^81^, suggesting the existence of a complex pathway that includes gene-environment modulation and environment/environment interactions. Finally other hypotheses are also relevant to be studied using potential interactions between 5HTTLPR and childhood trauma such as those concerning psychosis or suicidality in BD^17^,^83^.

## Conclusion

Gene-environment interactions represent a new challenge in psychiatric genetics^84^ and further studies of these complex interactions on clinical phenotypes are required to increase our understanding of the underlying etiological mechanisms. Such interactions are likely to occur when the effect of exposure to an environmental risk factor on a phenotype depends on the genotype. This study suggests a hypothetical model, in which emotional trauma and *SLC6A4* promoter variants interact to influence the AAO in BD. Further investigations in larger samples are required to test this hypothesis in order to help guide preventive strategies among subjects at risk of developing a BD (for example offspring of parents with BD and exposed to trauma).

## Additional Information

**How to cite this article**: Etain, B. *et al.* Interaction between *SLC6A4* promoter variants and childhood trauma on the age at onset of bipolar disorders. *Sci. Rep.* **5**, 16301; doi: 10.1038/srep16301 (2015).

## Figures and Tables

**Table 1:** Median (MAD) for AAO and CTQ total score according to *SLC6A4* genotypes.

Variables	*SLC6A4* Genotypes			P value
	L/L (N = 56)	L/S (N = 155)	S/S (N = 89)	
AAO (years)	20 (5)	22 (6)	22 (5)	0.48
CTQ total score	35.5 (6.5)	40 (8)	38 (8)	0.10

MAD: Median Absolute Deviation. AAO: age at onset. CTQ: Childhood Trauma Questionnaire. *SLC6A4*: serotonin transporter gene.

**Table 2:** Interaction between emotional neglect and *SLC6A4* genotypes on the AAO (initial sample).

	Beta	SE	t	P > t	[95% CI]	
LS	−0.011	0.007	−1.62	0.11	−0.025	0.002
SS	−0.017	0.007	−2.35	0.02	−0.032	−0.003
EN	−0.015	0.011	−1.32	0.19	−0.037	0.007
LS and EN	0.021	0.013	1.69	0.09	−0.004	0.047
SS and EN	0.037	0.014	2.62	0.009	0.009	0.065
intercept	0.220	0.006	38.05	0.000	0.209	0.232

EN: presence of emotional neglect. SE: standard error. CI: confidence interval of beta.

**Table 3:** Interaction between emotional neglect and *SLC6A4* genotypes on the AAO (replication sample).

	Beta	SE	t	P > t	[95% CI]	
SS	1.010	0.465	2.17	0.032	0.088	1.931
LS	0.491	0.556	0.88	0.38	−0.610	1.593
EN	0.595	0.465	1.28	0.20	−0.327	1.516
LS and EN	−0.953	0.539	−1.77	0.08	−2.022	0.115
SS and EN	−0.185	0.637	−0.29	0.77	−1.448	1.078
intercept	4.166	0.408	10.21	0.000	3.358	4.974

EN: presence of emotional neglect. SE: standard error. CI: confidence interval of beta.

**Table 4:** Interaction between emotional abuse and *SLC6A4* genotypes on the AAO (replication sample).

	Beta	SE	t	P > t	[95% CI]	
LS	0.954	0.349	2.73	0.007	0.262	1.647
SS	0.089	0.484	0.18	0.85	−0.870	1.048
EA	0.444	0.384	1.16	0.25	−0.318	1.206
LS and EA	−1.071	0.468	−2.29	0.024	−1.999	−0.142
SS and EA	0.159	0.593	0.27	0.79	−1.016	1.335
intercept	4.402	0.272	16.19	0.000	3.862	4.941

EA: presence of emotional abuse. SE: standard error. CI: confidence interval of beta.
